# On the Use of Chloroform and Ether at the Bellevue Hospital, N. Y.

**Published:** 1850-06

**Authors:** 


*On the use of Chloroform and Ether at the Bellevue Hospital, N. Y.* By D. Merdith Reese, M. D., Resident Physician.

The following is a portion of an article entitled "*Notes of Hospital Practice at Bellevue, N. Y.*," communicated for the *American Journal of Medical Sciences*, by Dr. Reese. It suggests a combination of Anæsthetic agents which merits the attention of the profession.

*Chloroform and Ether.*—The employment of these anæsthetic agents by inhalation has been signally successful in every department of hospital service. At first, and immediately after its introduction into the Masssachusetts General Hospital, the ether was used in all surgical operations of any magnitude, and in various painful and spasmodic diseases. No permanent evil effects followed, except in a single instance in which cerebral disease, probably of organic character, had pre-existed, and in this case the operation for the removal of extensive exostosis was necessarily tedious, and the inhalation had to be continued, and more than once repeated. The wound healed kindly, but the patient had to be subsequently sent to the insane department, and is deemed incurable. As, however, he had exhibited indications of insanity long prior to the inhalation, the mischief cannot fairly be ascribed to the etherization, although it would be indiscreet to employ this agent in any similar case. In no other instance did any evil happen which could be legitimately traced to the use of ether, although it was used very frequently for months, and until the introduction of *chloroform.*

This latter article was adopted as a substitute for the ether, as soon as authentic accounts were received of its employment by Dr. Simpson with success, and chiefly because we had found that the effects of ether were too slow in some cases, unequal in degree in others, while it occasionally failed.

The chloroform used at first was prepared by Dr. John Miller, then chemist and apothecary to the hospital, and was of entire purity and great strength. Our early experience with it developed two objections to its use; namely, it was sometimes too rapid in its action, occasionally cumulative, and deep anæsthesia came on too suddenly; hence we used it with great caution, and closely watched its effects. Anxious to avail ourselves of its greater certainty than ether, and yet avoid too deep anæsthetic effects, we determined to dilute the chloroform with ether, in the proportion of two measures of the latter, by weight, to one of the former. With this mixture, we have had every reason to be satisfied, and have hence very rarely employed the chloroform alone. The suggestion of this mode of dilution was first made by Dr. W. H. Van Buren, one of the visiting surgeons, and at his instance it was prepared and used for a surgical operation, with such entire success that we have employed this mixture ever since. In some instances, it is true, we have found patients so unsusceptible, that we have resorted to the chloroform alone, until the desired insensibility was produced, and then rendered the effect persistent, as long as necessary, by using the mixture of chloroform and ether, regarding it as a safer practice, cnd equally efficacious to this end.

Our method of employing these agents has been either by applying to the mouth and nostrils a hollowed sponge, moistened with ʒi or ʒij of chloroform, or ℥ss of the mixture; or, by means of a towel or handkerchief in lieu of the sponge, and which, on some accounts, is to be preferred. In no case, have we persisted continuously to apply either of these agents, so as to exclude the atmospheric air, but have always allowed the alternate inhalation of the air and that of the chloroform or ether. And our rule has been to remove the agent from the mouth as soon as anæsthetic effects have become manifest, reapplying the sponge or towel occasionally, if necessary. With these precautions, our experience has been so entirely free from any untoward or unpleasant consequences, that we can scarcely feel the force of the objections recently urged against anæsthetic agents, and are constrained to apprehend that the mischiefs described have resulted either from the want of due caution in their employment, or the lack of discrimination in the subjects, by overlooking existing pathological states which contraindicate their use. Certainly, we can have no sympathy with the surgeon whose unsuccessful operations are ascribed by himself to etherization; much less is any man authorized to attribute the want of success in another, to the fact that either chloroform or ether had been used in the case.

The following are a few of the specialities in which these agents have been employed here; namely:—

1st. In *reducing dislocations;* and here our experience has proved the invaluable importance of the discovery which has furnished to the profession these means of completely relaxing the muscles and ligaments of the larger joints. In several instances, luxations at the shoulder and hip of long duration, which, after repeated trials, could not be reduced by any amount of physical force applied in the ordinary way, were, after the inhalation of chloroform by the patient, found capable of ready reduction with the thumb and finger. If no other good were conferred by chloroform, this alone would render the discoverer a benefactor to the world.

2d. In hysterical, epileptic, and puerperal convulsions, we have had frequent opportunities of witnessing the speedy and effectual relief afforded by the inhalation of chloroform and ether; and, in the latter of these forms of disease, after extensive venesection and the other most potent antispasmodies had been tried in vain.

3d. In delirium tremens, we have frequently been able permanently to calm a violent patient, and induce profound and protracted sleep, after both lupuline and opium had failed.

4th. In paroxysmal or spasmodic asthma, a very few inhalations of ether or chloroform, without even approaching full anæsthesia, will be found more effectual for relief than any other remedy.

In tedious, protracted, or severe labour pains, and especially when malpresentation, or other causes, render painful operations necessary, whether manual or instrumental, the inhalation of chloroform, with the precautions already named, has been found to be uniformly safe, and pre-eminently useful, both to the mother and child; while it divests the most formidable operations of obsteteric surgery of their terrors, alike to the physician and his patient. We have not encouraged the use of this agency in natural labours, unattended by any considerable severity, although in these cases no evil has happened to mother or child in our hands. But, in all cases in which it has become important to lessen the sufferings of the mother, we have uniformly administered the chloroform, and have had every reason for undiminished confidence in its innocence and utility. Indeed, we had good ground to believe that, in several cases, our patient would have died undelivered, if we had been without chloroform; and yet, in more than one of these, the child as well as the mother was saved. Nor have we been able to detect, in a single instance, any subsidence or diminution of the labor pains, either in frequency or force, during the moderate degree of anæsthesia, which is all that is necessary for any useful purpose in obstetrical practice. And yet we have been obliged, in some severe labours, to continue the repetition of the inhalation during several hours—applying the chloroform to the nostrils and mouth for a few moments only, however, at the commencement of every pain—our patient importunately pleading for it, after having felt its efficacy in relieving her sufferings. Such has been our hospital experience in certainly one of the largest lying-in departments in our country.

*Surgical Operations.*—In all cases, when the severe or formidable operations of surgery have been called for, whether by the knife, saw, chisel, or red hot iron, we have not hesitated to give our patients the benefit of chloroform; and, in all this variety of infliction, we have witnessed entire immunity from pain, and in most cases there has been no consciousness of the operation. The limb has remained unheld and unmoved while the actual cautery has been made repeatedly to traverse a joint; and, in one instance of the amputation of a thigh, the patient remained ignorant of his mutilation until the fifth day, when it became necessary to dress the stump, he all the while supposing that his leg was bound up in splints, and complaining of that very common, though imaginary, sensation in the toes of the amputated limb. The success of operations, and the early healing of the wounds thus made, have in no instance been hindered or delayed, so far as we could perceive; and we have even surmised the contrary. In primary amputations, after compound or comminuted fractures, when these are judged necessary, the additional shock to the nervous system, so much dreaded, is in a great measure obviated by the judicious use of chloroform. And, whatever may be the prejudice existing or engendered by panic-makers, who are for the most part mere theorists, the extensive experience and success of practical men, here and elsewhere, should disabuse the profession and the public of the erroneous views which have been promulgated of late, on the basis of a few unsuccessful and probably indiscreet experiments, and isolated cases of unfortunate termination. The profession should not only allow their patients the advantages derived from this new agency to protect them from suffering, but they should feel that they are not at liberty to hazard the safety of their patients by withholding from sufferers of every class the benefit of anæsthesia, now that science has conferred this boon upon afflicted humanity.

